# Changes in Boston

**Published:** 1855-03

**Authors:** 


*Changes in Boston.*—Dr. Geo. S. Jones has resigned as associate editor of the Boston Medical Journal. His place is taken by Drs. Wm. W. Morland and Francis Minot. The new editors send out a graceful salutatory. The Boston Journal has, until recently, been the only weekly in the country, and has enjoyed a large circulation. Aside from a fault of publishing all communications sent, whether good, bad, or indifferent, and a more serious error in an indiscriminate praise of all books sent to it by publishers, it has ever been a readable and profitable journal. We mention the character of its reviews in no spirit of unkindness, but in the hope that the new editors may avoid such an inconsistency as their first number contains in the form of a vigorous puff of Dr. Holmes' really excellent pamphlet on puerperal fever, which was preceded only a few weeks ago by an equally decided approval of the precisely opposite doctrines advanced by Dr. Meigs in his work on childbed fevers.

Aside from these changes editorial, some changes professional have taken place in the time-honored Medical Department of Harvard University. Dr. David Storer succeeds Dr. Walter Channing in the professorship of Obstetrics and Medical Jurisprudence, and Dr. Edward Hammond Clarke succeeds Dr. Jacob Bigelow in the chair of Materia Medica. A professorship of Clinical Medicine is substituted for the present Lectureship, and George Cheyne Shattuck, M. D., is to be the first professor. These are important changes in an important and influential school. H.

